# Improving attendance in addictions - do quality improvement plans work?

**DOI:** 10.1192/bjo.2021.552

**Published:** 2021-06-18

**Authors:** Soraya Mayet, Shumaila Shahbaz

**Affiliations:** Humber Teaching Foundation Trust

## Abstract

**Aims:**

We assessed whether a quality improvement plan initiated in 2018 had sustained benefits for improving attendance rates at addiction prescriber reviews, after 13 months.

**Method:**

The QIP re-audit had Humber Teaching NHSFT approval. We assessed electronic healthcare records of patients prescribed OST at a specialist addictions service, spanning a large geographical area, split into three Hubs. Data were analysed via Microsoft excel.

Baseline data for the whole addictions service were collected in April 2018 (n = 343), followed by QIP implementation. The QIP included a new appointment letter explaining the importance of the prescriber review, text message confirmation and reminder the day before, verbal reminder from keyworker and a call from the prescriber explaining the importance of attending (for persistent non-attenders). In the event of nonattendance, a medication safety review was completed. Further data were collected in December 2018 (n = 339) and a re-audit of one Hub (n = 91) was completed in Jan 2020.

**Result:**

At baseline in April 2018, half (50% n = 170/343) of all patients had attended an addictions prescriber review in the last 3 months; Hub 1 (55%; n = 52/95), Hub 2 (34%; n = 45/133) and Hub 3 (65%; n = 73/115). The Quality Improvement Plan was implemented. Attendance rates for subsample (Hub 1) conducted in Oct 2018 showed a reduction in attendance (51%; n = 48/92). This led to the enhanced Quality Improvement Plan.

After the enhanced Quality Improvement Plan implementation in Dec 2018, attendance rates improved for all Hubs to 76% (n = 258/339); Hub 1 (77%; n = 72/93), Hub 2 (73%; n = 97/133), Hub 3 (79%; n = 89/113). For non-attending patients, a medication review was conducted in their absence by the prescriber for most (94%; n = 74/81) patients (see table 1 and Figure 1).

In January 2020, reassessment of attendance rates for Hub 1 (subsample), in January 2020 (n = 91) which showed attendance had increased to 86% (n = 78/91). All (100% n = 13) patients who did not attend for the prescriber review in person, had a medication review in their absence. In addition, the reasons for nonattendance were discussed with the patient and their keyworker, following which they were booked for a subsequent appointment.

**Conclusion:**

Nonattendance at clinical appointments causes a significant financial burden across the NHS. It was fantastic to see that the QIP improved patient attendance rates and this was sustained and improved, over a year later. Serial non-attenders may need an enhanced strategy.

